# Second Wave of COVID-19 Pandemic in Argentinian Population: Vaccination Is Associated With a Decrease in Depressive Symptoms

**DOI:** 10.3389/fpsyt.2022.832352

**Published:** 2022-06-23

**Authors:** Pedro Benedetti, Alejo Barbuzza, Franco Moscato, Victoria Reppucci, Celina Goyeneche, Cynthia Katche, Jorge H. Medina, Haydee Viola, Fabricio Ballarini, Diego Moncada

**Affiliations:** ^1^Instituto Tecnológico de Buenos Aires, Buenos Aires, Argentina; ^2^Facultad de Medicina, Instituto de Biología Celular y Neurociencia “Prof. E. De Robertis” (IBCN), UBA-CONICET, Buenos Aires, Argentina; ^3^Departamento de Fisiología, Facultad de Ciencias Exactas y Naturales, Biología Molecular y Celular “Dr. Héctor Maldonado” (FBMC), University of Buenos Aires (UBA), Buenos Aires, Argentina; ^4^Centro Integrativo de Biología y Química Aplicada, Universidad Bernardo O'Higgins, Santiago, Chile

**Keywords:** COVID-19, GAD-7, PHQ-9, vaccination, physical activity

## Abstract

**Background:**

Since the irruption of the coronavirus disease 2019 (COVID-19) the planet has submerged in a time of concern and uncertainty, with a direct impact on people's mental health. Moreover, the recurrent outbreaks that periodically harry different regions of the world constantly refocus people's concerns to the pandemic. Yet, each new wave heats the diverse countries in different situations, including the advances in their vaccination campaigns. In this research, we studied the levels of the general anxiety disorder (GAD) and depression in the Argentine population across the first and second waves of infections that occurred in our country.

**Methods:**

We conducted an on-line survey, within each peak of the pandemic. People were asked to self-report GAD and depression symptoms using the GAD-7 and PHQ-9 questioners, inform their vaccination status, the frequency they performed physical activity as well as working condition and modality. Here, we identified the more vulnerable groups and evaluated factors that could mitigate the rise of these mental disorders, focusing on vaccination.

**Results:**

Our data shows that reported GAD and depression levels were higher during the second wave than during the first one. More importantly, vaccinated people were less depressed than non-vaccinated people, while GAD levels remained equivalent in both groups. Other factors directly associated with lower GAD and depression levels were performing frequent physical activity and being employed, regardless of the employment modality. These observations were replicated in different age ranges and genders.

**Conclusion:**

This work evidences GAD and depression in different pandemic waves in Argentina, as well the factors that may contribute to reducing the magnitude of these disorders, including vaccination.

## Introduction

COVID-19 is a disease caused by the coronavirus SARS-CoV-2 that has stroked mankind for more than 18 months. So far, up to the end of August 2021 it has caused 4.4 million deaths on the planet and 110.000 originated in Argentina, which unfortunately ranks 15th in the world (1). In addition to the many physical illnesses associated with the COVID-19, it also causes psychological disorders such as depression and anxiety. Several examples of this have been reported around the world (2–11). In this work, we studied the self-perceived levels of GAD and depression in adults between 18 and 50 years old, along the pandemic period, throughout two cross-sectional surveys performed in the first (November 2020) and at the second waves (May 2021) of the SARS-CoV-2, in the Buenos Aires Metropolitan Area of Argentina. Furthermore, our objective was to analyze the impact of vaccination, physical activity and work modality, under the hypothesis that they may act as possible protective factors of the population's mental health after such a prolonged period of the pandemic.

It is reported that key changes in life domains, including home confinement, reduction in face-to-face social interaction and disrupted occupation/ education roles, are associated with the impairments observed in common mental disorders such as anxiety and depression (12). The clearer tool available to end such disrupted daily routines is massive vaccination at the national and worldwide level. Fortunately, different COVID-19 vaccines were developed and proved efficient to reduce the number of hospitalizations with severe symptoms and the number of casualties. Unfortunately, there is a continuum from complete acceptance to total refusal of all vaccines, with vaccine hesitancy lying between the two poles (13). One of the reasons to refuse vaccination is the fear of vaccines side effects (14). Therefore, adequate information on this subject is critical to make people aware about the importance of weighing their decision to accept and foster the vaccination process.

The interaction between mental disorders and the effect of vaccination is intricate. It was reported that GAD and depression can increase associated with vaccine hesitancy (15, 16), but also people with higher levels of anxiety are those who agree to be vaccinated (15, 17). On the other hand, these psychological factors negatively influence vaccine efficacy (18). In Argentina the first wave of COVID-19 occurred in the absence of vaccines (November 2020), but the second wave (May 2021) surprised the country in the middle of the massive vaccination campaign which to that moment vaccinated more than a million people per week (19). Thus, we registered the self-reported levels of GAD and depression in the adult population during these two waves of COVID-19 and analyzed the impact of vaccination on their mental health.

Besides vaccination, other public policies were applied during the pandemic for the prevention of infections, which included social isolation. Elevated self-reported levels of anxiety and depression were associated with self-reported COVID-19 pandemic-related self-isolation and self-quarantine activity (20–22). One of the consequences of social isolation was the obligation or necessity of many people to carry out their work at home. In this study we have analyzed the levels of GAD and depression in adults in relation to their working status (workers or non-workers) and modality (face-to-face, work from home or hybrid). Finally, a behavioral factor that contributes to reduce the risk of suffering from these ailments is the practice of physical activity. Various studies have been carried out relating the frequency and intensity of physical activity with respect to its effectiveness in terms of mental health (23–26). Here, we determined the distribution of the adult population that exercises with low or high weekly frequency and analyzed its relation with people's self-perception of GAD and depression.

In summary, we conducted the research under the hypothesis that vaccination, physical activity and work modality, may act as possible protective factors on GAD and depression during the pandemic waves. Our results show variations of GAD and depression reported at the population level in two consecutive COVID-19 outbreaks. They describe the impact in different age groups and genders, and shed light on the positive effects of vaccination, physical activity and working status on the mental health during the pandemic.

## Materials and Methods

### Study Settings and Participants

A cross-sectional design was performed to survey adult population, residents of the Metropolitan Area of Buenos Aires (AMBA), Argentina, in two different moments during the COVID-19 pandemic. The participants were recruited via social media of local scientific communicators, and responded to the survey through the platform Google Forms. In both surveys, the participants completed the GAD-7 and the PHQ-9 tests and reported their COVID-19 vaccination status, the weekly frequency they performed physical activity, their employment status, and the work modality (Supplementary Materials). After accessing the webpage, the participants were allowed to complete the survey without time limits. In general, this operation lasts approximately 15 min. Only full answered surveys were considered for the analysis.

Data were collected first in October/November 2020 (from 22nd October 2020 to 7th November 2020, *n* = 1,531, 79,29% women) and second in May 2021 (from 5th May 2021 to 10th May 2021, *n* = 4,576, 83,10 % women). We divided this population into two groups of study: people between 18 and 30 years old and people between 31 and 50 years. This division was performed using as guidance the one established by the national institute for statistics and census (INDEC) to differentiate between young (15–29 years) and adults (17, 26, 30–58) within the economically active population (27–30). In our case we excluded the population under 18 years old due to the impossibility of obtaining reliable on-line informed consents signed by the parents of the minors. These groups can share certain lifestyles, and characteristics: young are studying or having a low-responsibility jobs, in our country a large percentage of them live with their parents (64%) and do not have children (90%) (29). On the other hand, the majority of people between 30 and 60 years of age must have greater family care responsibilities and are the economic support of the family (28–30).

In total 2,830 people responded the first survey. We excluded from the analysis 1,285 respondents that did not belong to the AMBA region and 14 for being older than 50 years old. In the first case exclusions were performed for not being representative samples of their geographical regions. In the second for not being representative of the age range (>50) of the target region. In the second survey responded 7,735 participants. In this case, and to match samples, 3,002 were excluded for geographical reasons and 157 for being older than 50 years old. People younger than 18 years were blocked by the system. Considering the AMBA population and the proportion of people between 18 and 50 years old, at least of 379 persons are required to have a confidence level of 95% with a 5% of margin error. Since we surveyed people using an online convenience method, we decided to maximize the sample size to all the participants that responded within the data collection period.

The AMBA is the biggest urban conglomerate in Argentina. It is a geographical region composed of the Autonomous City of Buenos Aires and multiple political units of the Buenos Aires province with a population of approximately 15 million people.

### Dynamic of COVID-19 Pandemic and Vaccination in Argentina

In Argentina, the first case of COVID-19 was detected on the 3rd March 2020. On the 18th March 2020 the government decreed a nationwide lockdown (31) that lasted until the 7th November. During this period, only the essential activities were permitted. Restrictions were revised and updated every 2 weeks by a phase system that moved depending on the epidemiological indicators.

The surveys were conducted within the first and the second waves of contagions, in the context of particular epidemiological situations. The first one, performed in October/November 2020, matched with the end of the first wave of SARS-CoV-2 when the total confirmed cases in Argentina were 1.236.851, with 33.348 confirmed deaths, a 59,6% of occupied Intensive Therapy Unit (ITU) beds in the AMBA (32), and without vaccines available. At the end of the second data recollection (10th May 2021) Argentina counted 3.165.121 total confirmed cases, 67.821 confirmed deaths, and an ITU occupancy of 77% in the AMBA. However, at this moment 7,718,272 people were vaccinated with one dose (17.2%) and 1,404,487 with two doses (3.1%), in the middle of a nationwide vaccination campaign (33). The first vaccinated group was the risk population (Sanitary personnel, people aged over 60 years, and those with certain preexistent medical problems).

### Survey Structure Measures

#### Socio-Demographics

in both surveys, all participants (October/November 2020 and May 2021) informed their gender (“men,” “women,” and “other”), age (“18” up to “50”), area of residence (“AMBA” or “Not AMBA”), employment status (“worker” or “non-worker”), work modality (“Face to face,” “Work from home” or “Hybrid”), and number of days they performed physical activity per week (“0” up to “7”). In the second survey, we also asked about the vaccination status (vaccinated, not vaccinates or I'd rather not answer); 3 participants decided not to answer and were excluded from this part of the analysis. Only people of the AMBA region were considered for this study.

### Mental Health Measures

#### Generalized Anxiety

Generalized anxiety was measured through the 7-item Generalized Anxiety Disorder Scale (GAD-7, 34) which is validated and widely used in various populations (35, 36). This mental health instrument gathers information about generalized anxiety symptoms of the 2 weeks previous to the questionnaire. Respondents report their symptoms using a 4-point Likert rating scale ranging from 0 (not at all) to 3 (almost every day) along 7 questions, therefore the total score ranges from 0 to 21. Scores of 0–4 are thought to represent minimal anxiety, 5–9 mild anxiety, 10–14 moderate anxiety, and 15–21 severe anxiety (34). We assessed the reliability in both periods of data collection by calculating the Cronbach indexes, which were contained within the 95% of confidence interval (CI). They were α = 0.88 (CI 0.87–0.89) for the first wave and α = 0.89 (CI 0.885–0.895) for the second one, reflecting a high reliability.

#### Depression

Depression was measured using the Patient Health Questionnaire (PHQ-9; 37). The PHQ-9 resulted in a reliable and widely validated measure of depressive symptoms (37–39). Each respondent must answer nine questions that describe depression symptoms, considering the last 2 weeks. Each question can be answered with a 4-point Likert rating scale ranging from 0 (not at all) to 3 (almost every day) along nine questions, thus the total score ranges from 0 to 27. Scores of 0–4 suggest minimal depression, 5–9 mild depression, 10–14 moderate depression, 15–19 moderately severe depression, and 20–27 severe depression (37). The Cronbach indexes were α = 0.86 (CI 0.849–0.87) for the first wave and α = 0.86 (CI 0.854–0.866) for the second one, reflecting high reliability on the data collected in both periods.

### Ethical Considerations

This study was approved by ethics council of the Life Sciences Department of the Instituto Tecnológico de Buenos Aires. Before answering the survey, each participant was provided with an informed consent that had to be approved to participate in the study. Data was analyzed to maintain anonymity of the participants. All the procedures conducted in this study followed the ethical standards of the institutional and/or national research committee as well as with the Helsinki Declaration of 1975, as revised in 2008.

### Statistical Analysis

Depending on the type of variable we calculated descriptive statistics for the sample. For each continuous variable (GAD-score from GAD-7, Depression score from PHQ-9 and mean day of weekly days of physical activity) descriptive statistics were expressed as means with standard error of the mean (SEM) and for non-continuous variables as counts and percentages (%). The specific statistical tests used in each case are informed in the corresponding figure legends. Descriptive statistic for each figure is supplied as Supplementary Tables 1–6. Normality and homocedacy were analyzed using the Kolmogorov-Smirnov, Bartlett's and F tests (Supplementary Tables 7–12). Non-parametrical tests were used to analyze samples that did not follow a normal distribution or the homocedacy requirements of parametric tests. Outliers were searched using the ROUT method with a Q value of 1%. All the non-parametric statistics were re-analyzed using the equivalent parametric test by assuming a normal distribution of the means due to the large sample size. No differences between tests were found. The differences were considered significant when p < 0.05 (α = 0.05). We report exact *p*-values, no adjustments were adopted. The statistical analysis was performed using GraphPad Prism^®^ 8.0.1 software. Effect sizes are reported for all the significant differences, in the correspondent figure legends. For this purpose, Cohen's d was calculated for *t*-tests; Cohen's f^2^ and η^2^ for one-way ANOVAs and Cohen's f^2^ and partial η^2^ ($\eta_{\text{p}}^{2}$) for two-way ANOVAs. Cohen's d and f^2^ were calculated using the WebPower On-line software; η^2^ and $\eta_{\text{p}}^{2}$ were calculated manually. In both cases, we assumed a normal distribution of the sample mean, due to its large size, to perform the calculations. Effect sizes for X^2^ independency tests are expressed as phi (φ) coefficient. The relation between GAD and depression scores were calculated using the Spearman's rank correlation coefficient (r_s_).

## Results

**Figure 1:**
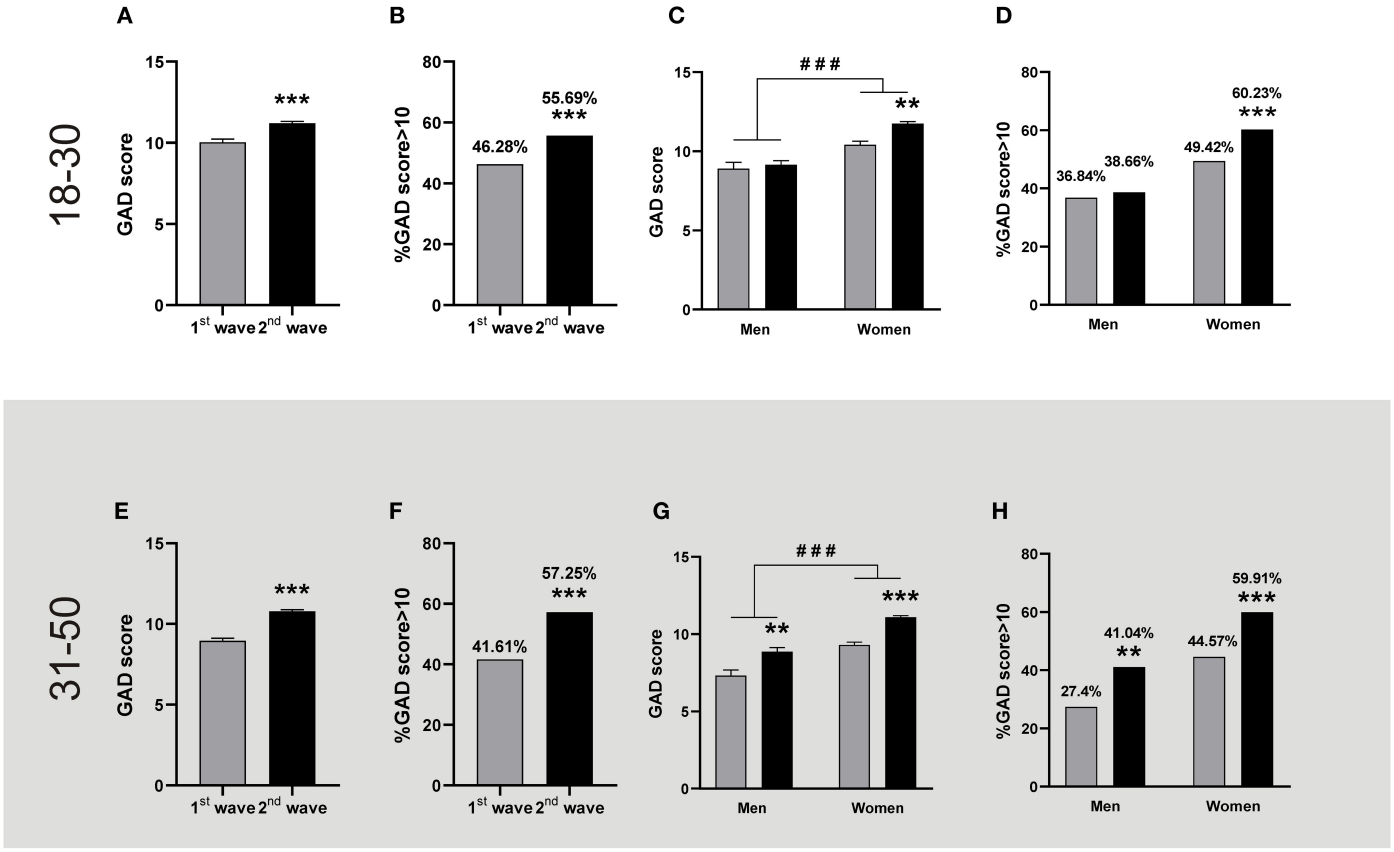
GAD levels registered during the first and the second wave of COVID-19. The figures show the mean + SEM GAD scores or the percentage of people with GAD scores >10 during the first (grey bars) and the second (black bars) wave. The descriptive statistics are reported in Supplementary Table 1. **Top panel:** People aged between 18 and 30. **(A)** People reported higher GAD scores in the 2nd wave (*n* = 1,844) than in the 1st wave (*n* = 685). Mann-Whitney *U*-test, ^***^p < 0.001; Cohen's *d* = 0.23. **(B)** The percentage of people with a GAD score >10 was higher during the second wave; ^***^p < 0.001, X^2^, φ = 0.096. **(C)** Women but not men reported higher GAD scores in the 2nd wave. ^###^*p* < 0.001 women vs. men, and ^**^p < 0.01 vs. 1st wave after two-way ANOVA; Cohen's *f*^2^ = 0.23; $\eta_{\text{p}}^{2}$ = 0.023 for the gender factor and $\eta_{\text{p}}^{2}$ = 0.003 for wave factor (women: 1st wave *n* = 514, 2nd wave *n* = 1,456. Men: 1st wave *n* = 171, 2nd wave *n* = 388). **(D)** A higher percentage of women reported a GAD score >10 during the 2nd wave; ^***^p < 0.001 vs. 1st wave, X^2^, φ = 0.10. **Bottom panel:** People aged between 31 and 50. **(E)** People reported higher GAD scores in the 2nd wave (*n* = 2,732) than in the 1st wave (*n* = 846). Mann-Whitney *U*-test, ^***^p < 0.001, Cohen's *d* = 0.37. **(F)** The percentage of people with a GAD score >10 was higher during the second wave; ^***^p < 0.001, X^2^, φ = 0.13. **(G)** Both genders reported higher GAD scores in the 2nd wave. ^###^*p* < 0.001 women vs. men, and ^**^p < 0.01 and ^***^p < 0.001 vs. 1st wave after two-way ANOVA; Cohen's f^2^ = 0.49 and 0.31 for men and women respectively; $\eta_{\text{p}}^{2}=$0.016 for the gender factor and $\eta_{\text{p}}^{2}$ = 0.010 for wave factor (Women: 1st wave *n* = 700, 2nd wave *n* = 2,347. Men: 1st wave *n* = 146, 2nd wave *n* = 385). **(H)** A higher percentage of women and men reported GAD scores >10 during the 2nd wave; ^**^p < 0.01 and ^***^p < 0.001 vs. 1st wave, X^2^., φ = 0.13 for both men and women.

We started by analyzing the GAD in people from 18 to 30 years old (from now young adults) and observed increased scores during the second wave compared to the first wave (Figure 1A). In fact, mean GAD scores during this second wave went over 10, usually considered the cut-off between mild and moderate GAD conditions (34). Hence, we analyzed whether the increased anxiety was reflected by change in the percentage of people expressing moderate to severe GAD (score>10) and we observed that, during the second wave of the pandemic, the population with these conditions increased by almost 10% (Figure 1B). A posterior gender analysis revealed a differential effect of the wave on young men and women. The latter group reported higher GAD scores during the second outbreak, and in addition women presented higher GAD than men in both waves (Figure 1C). Actually, while the man population with moderate to severe GAD increased less than 2% during the second wave, that of the woman population raised almost 11% (Figure 1D).

Then, we evaluated the same variables for people between 31 and 50 years old (from now adults). In this case, we also observed a higher mean GAD scores in the second wave of contagion than in the first one, which overpassed the value of 10 (Figure 1E). This increase also reflected a rise of more than 15 % of the population that reported moderate to severe GAD scores during this period (Figure 1F). The posterior gender analysis revealed that women were more anxious than men since the first wave; in addition, this gender difference remained during the rise of anxiety observed in both genders at the second wave (Figure 1G). In particular, population with moderate to severe GAD increased around 15% in adult men and women, showing that both genders contributed evenly to the rise of GAD observed in adult people during the second wave (Figure 1H). It is worth noting that during the second wave, for both age ranges, circa 60% of women reported moderate to severe GAD while only 40 % of men were in these conditions.

**Figure 2:**
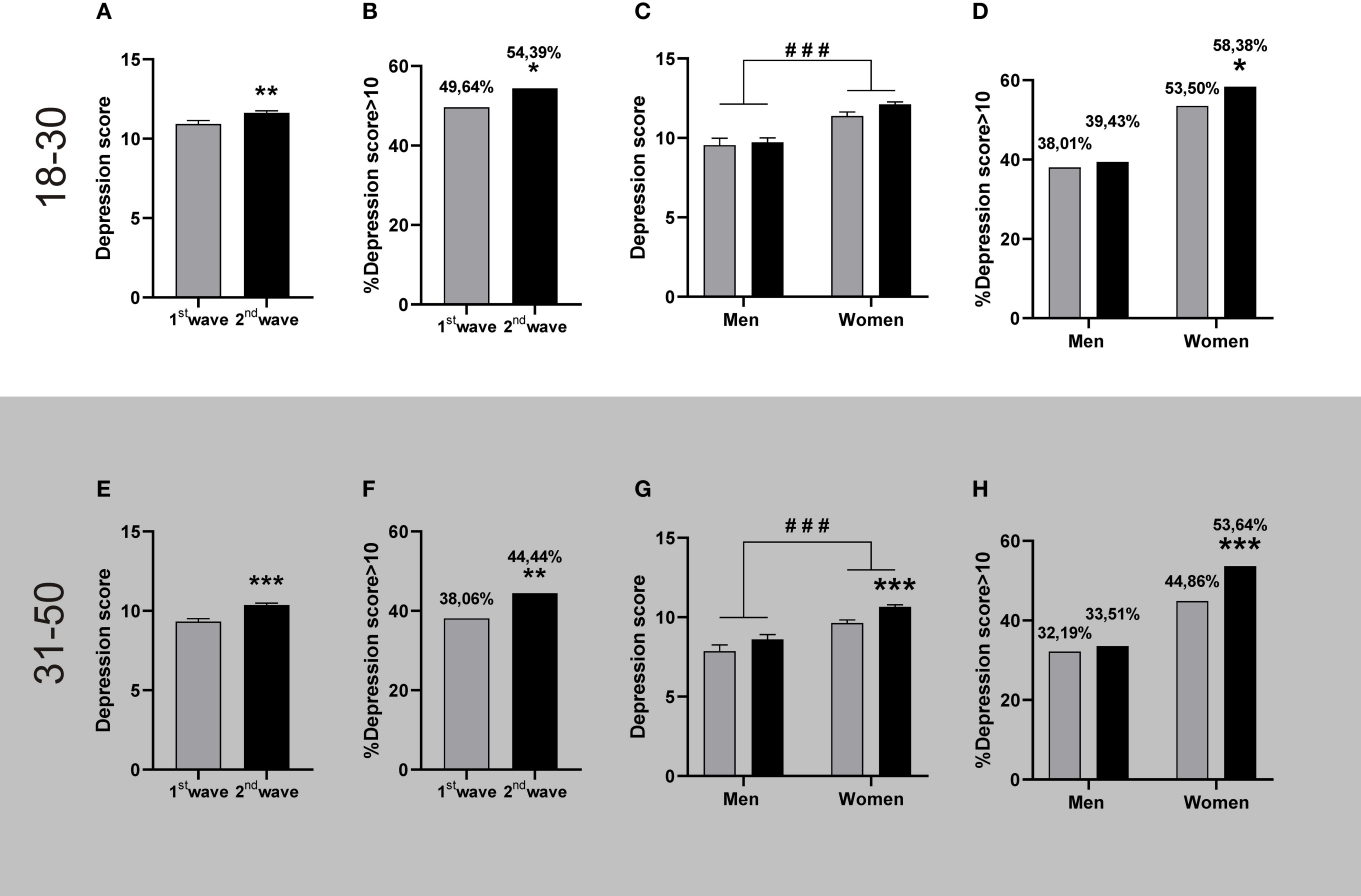
Depression levels registered during the first and the second wave of COVID-19. The figures show the mean + SEM depression score or the percentage of people with depression scores >10 during the first (grey bars) and the second (black bars) wave. The descriptive statistics are reported in Supplementary Table 2. **Top panel:** People aged between 18 and 30. **(A)** People reported higher depression scores during the 2nd wave (*n* = 1,844) than in the 1st wave (*n* = 685). Mann-Whitney *U*-test, ^**^p < 0.01; Cohen's *d* = 0.11. **(B)** More people presented depression scores >10 during the 2nd wave; ^*^p < 0.05, X^2^, φ = 0.042. **(C)** Both genders reported equivalent GAD levels in both waves, and women reported higher GAD than men within each wave, ^###^*p* < 0.001 women vs. men, after two-way ANOVA; $\eta_{\text{p}}^{2}$ = 0.018 for the gender (Women: 1st wave *n* = 514, 2nd wave *n* = 1,456. Men: 1st wave *n* = 171, 2nd wave *n* = 388). **(D)** A higher percentage of women reported depression scores >10 during the 2nd wave; *p < 0.05 1st wave, *X*^2^, φ = 0.043 for women. **Bottom panel:** People aged between 31 and 50. **(E)** People reported higher depression scores during the 2nd wave (*n* = 2,732) than in the 1st wave (*n* = 846). Mann-Whitney *U*-test, ^***^p < 0.001; Cohen's *d* = 0.19. **(F)** More people presented depression scores >10 during the 2nd wave; ^**^p < 0.01, X^2^, φ = 0.054. **(G)** Women reported more depression in the second wave. ^###^*p* < 0.001 women vs. men, and ^***^p < 0.001 vs. 1st wave, after two-way ANOVA; Cohen's f^2^ = 0.17 for women; $\eta_{\text{p}}^{2}$ = 0.016 for the gender factor and $\eta_{\text{p}}^{2}$ = 0.010 for wave factor. (Women: 1st wave *n* = 700, 2nd wave *n* = 2,347. Men: 1st wave *n* = 146, 2nd wave *n* = 385). **(H)** A higher percentage of women presented depression scores >10 during the 2nd wave; ^***^p < 0.001 vs. 1st wave, *X*^2^, φ = 0.073 for women.

In young adults, the depression scores reported during the second wave were significantly higher than in the first one (Figure 2A). This was also reflected in a higher percentage of people reporting moderate to severe depression symptoms during the second wave with respect to the first one, representing approximately 5% more of the surveyed population (Figure 2B). The posterior gender analysis revealed that young women reported higher depression levels than men in both outbreaks (Figure 2C). Analyzing the percentage population that reported moderate to severe symptoms, we observed a significative increase of almost 5% in women during the second outbreak (Figure 2D). A similar profile was observed in adults, with higher depression levels reported during the second wave (Figure 2E); reflecting an increase of 6 % in the population with moderate to severe depression (Figure 2F). As with the young adults, women reported higher depression scores than men in both waves. Women, also reported higher depression scores during the second outbreak (Figure 2G), a moment when the percentage of female population with moderate to severe symptoms increased by 9% (Figure 2H). On the contrary, the adult male population reported similar levels of depression during these pandemic waves, which was reflected in similar percentages of the adult male population with depression scores over 10 in both waves (Figures 2C,D,G,H).

Given the well-known comorbidity between GAD and depression, we evaluated their relation in both outbreaks and observed that their scores correlated positively for both age ranges and almost indistinguishable in both waves (Young adults r_s_ = 0.73 (95% CI: 0.6895–0.7623) and r_s_ = 0.69 (95% CI: 0.6665–0.7156) for 1st and 2nd wave, respectively. Adults: r_s_ = 0.68 (95% CI: 0.6410–0.7158) and r_s_ = 0.72 (95% CI: 0.6998–0.7371) for 1st and 2nd wave, respectively). Neither the slope of the linear regressions adjusting to the correlations nor the basal values changed between outbreaks (*p* > 0.05), pointing to an equivalent relation between GAD and depression of the populations surveyed in the first and the second outbreak.

**Figure 3:**
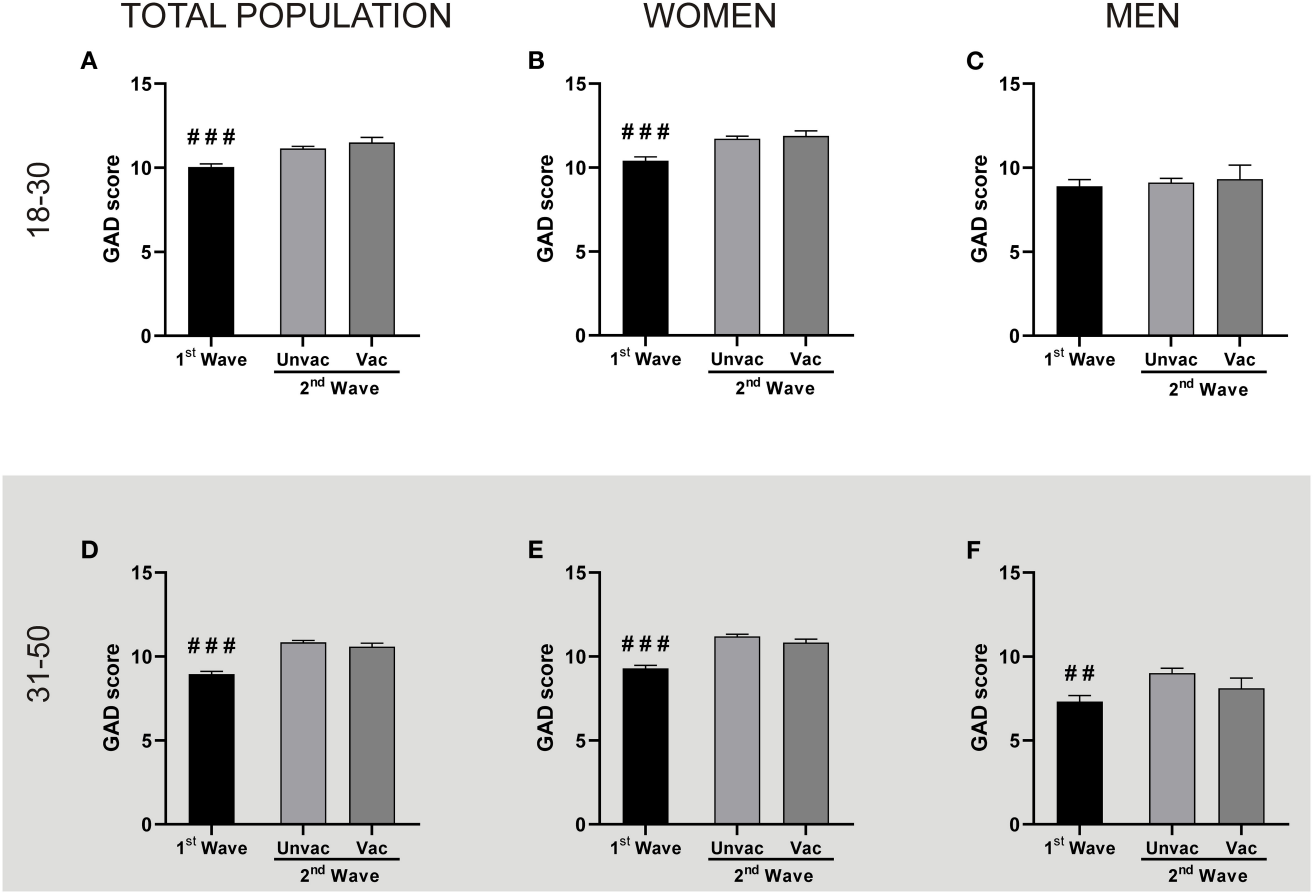
The vaccination status did not correlate with GAD levels changes. Each panel compares the GAD scores reported by the unvaccinated people during the 1st and the 2nd wave, and the GAD scores between unvaccinated (Unvac) and vaccinated (Vac) people within the 2nd wave, by two independent Mann-Whitney *U*-test. Data shown as mean + SEM. The descriptive statistics are reported in Supplementary Table 3. **Top panel:** People aged between 18 and 30. **(A)** Total population: ^###^*p* < 0.001 vs. unvaccinated; Cohen's *d* = 0.21; (Unvac 1st wave *n* = 685; unvaccinated *n* = 1,538; vaccinated *n* = 303). **(B)** Women: ^###^*p* < 0.001 vs. unvaccinated; Cohen's *d* = 0.26 (1st wave *n* = 514; unvaccinated *n* = 1,195; vaccinated *n* = 259). **(C)** Men: (1st wave *n* = 171; unvaccinated *n* = 343; vaccinated *n* = 44). **Bottom panel:** People aged between 31 and 50. **(D)** Total population: ^###^*p* < 0.001 vs. unvaccinated; Cohen's *d* = 0.38 (Unvac 1st wave *n* = 846; unvaccinated *n* = 2,035; vaccinated *n* = 692). **(E)** Women: ^###^*p* < 0.001 vs. unvaccinated; Cohen's *d* = 0.39 (1st wave *n* = 700; unvaccinated *n* = 1,709; vaccinated *n* = 633). **(F)** Men: ^##^*p* < 0.01 vs. unvaccinated; Cohen's *d* = 0.40 (1st wave *n* = 146; unvaccinated *n* = 326; vaccinated *n* = 59).

**Figure 4:**
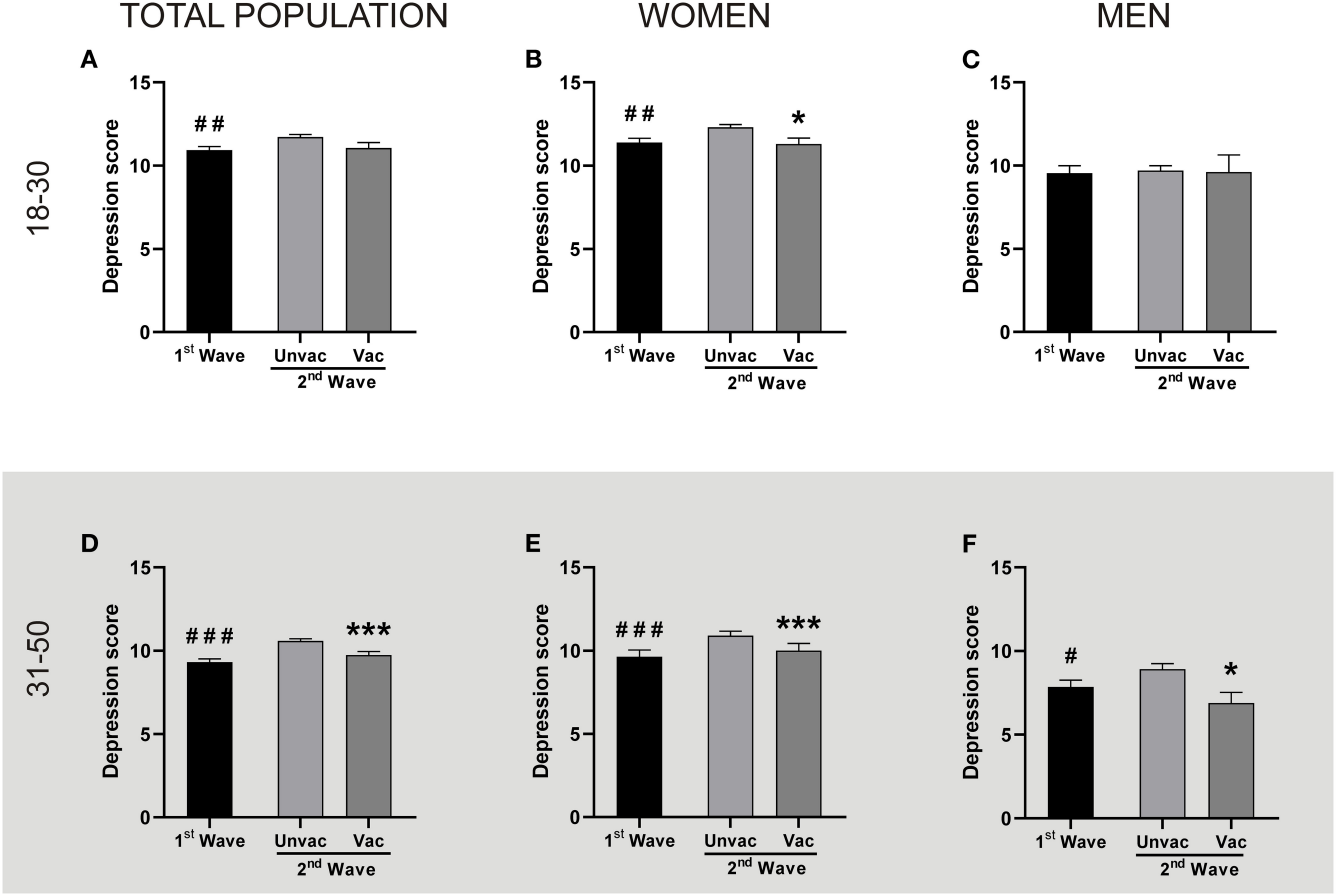
Vaccination was associated with lower depression levels in adult people and young adult women. Each panel compares the Depression levels reported by the unvaccinated people during the 1st and the 2nd wave, and also the same parameter between unvaccinated and vaccinated people within the 2nd wave, by two independent Mann-Whitney *U*-test. The descriptive statistics are reported in Supplementary Table 4. **Top panel:** People aged between 18 and 30. **(A)** Total population: ^##^*p* < 0.01 1st Wave (*n* = 685) vs. Unvaccinated (*n* = 1,538); Cohen's *d* = 0.13. Vaccinated (*n* = 303). **(B)** Women: ^##^*p* < 0.01 and ^*^p < 0.05 vs. Unvaccinated; Cohen's *d* = 0.16 and 0.17 respectively (1st wave *n* = 514; unvaccinated *n* = 1,195; vaccinated *n* = 259). **(C)** Men: (*n* = 171; 343; 44, for 1st wave, unvaccinated and vaccinated, respectively). **Bottom panel:** People aged between 31 and 50. **(D)** Total population: ^###^*p* < 0.001 and ^***^p <0,001 vs. unvaccinated; Cohen's *d* = 0.22 and 0.15 respectively (1st wave *n* = 846; unvaccinated *n* = 2,035; vaccinated *n* = 692). **(E)** Women: ^###^*p* < 0.001 and ^***^p < 0.001 vs. unvaccinated; Cohen's *d* = 0.23 and 0.16 respectively (1st wave *n*=700; unvaccinated *n* = 1,709; vaccinated *n* = 633). **(F)** Men: ^#^*p* < 0.05 and *p < 0.05 vs. unvaccinated; Cohen's *d* = 0.20 and 0.38 respectively (1st wave *n* = 146; unvaccinated *n* = 326; vaccinated *n* = 59).

It is worth notice that, while vaccines were unavailable in Argentina during the first wave, the second wave started during the vaccination campaign. Thus, we also studied whether the vaccination status was related to GAD and depression scores. We observed that regardless of the vaccination status, GAD scores during the second wave were higher than during the first wave, both, for young adult (Figure 3A) and adult (Figure 3D) populations, with the exception of young adult men (Figures 3B,C,E,F). In the same direction, the unvaccinated people from the second wave reported higher depression scores than the group of the first wave (Figure 4, except for young men Figure 4C). However, those persons of the adult population that received at least one dose of any COVID-19 vaccine reported fewer depression symptoms than those unvaccinated in the second wave (Figures 4D,E,F). This same effect repeated in the group of young adult women (Figure 4B). Thus, excepting the young adult men group, vaccination was associated with lower depression symptoms.

**Figure 5:**
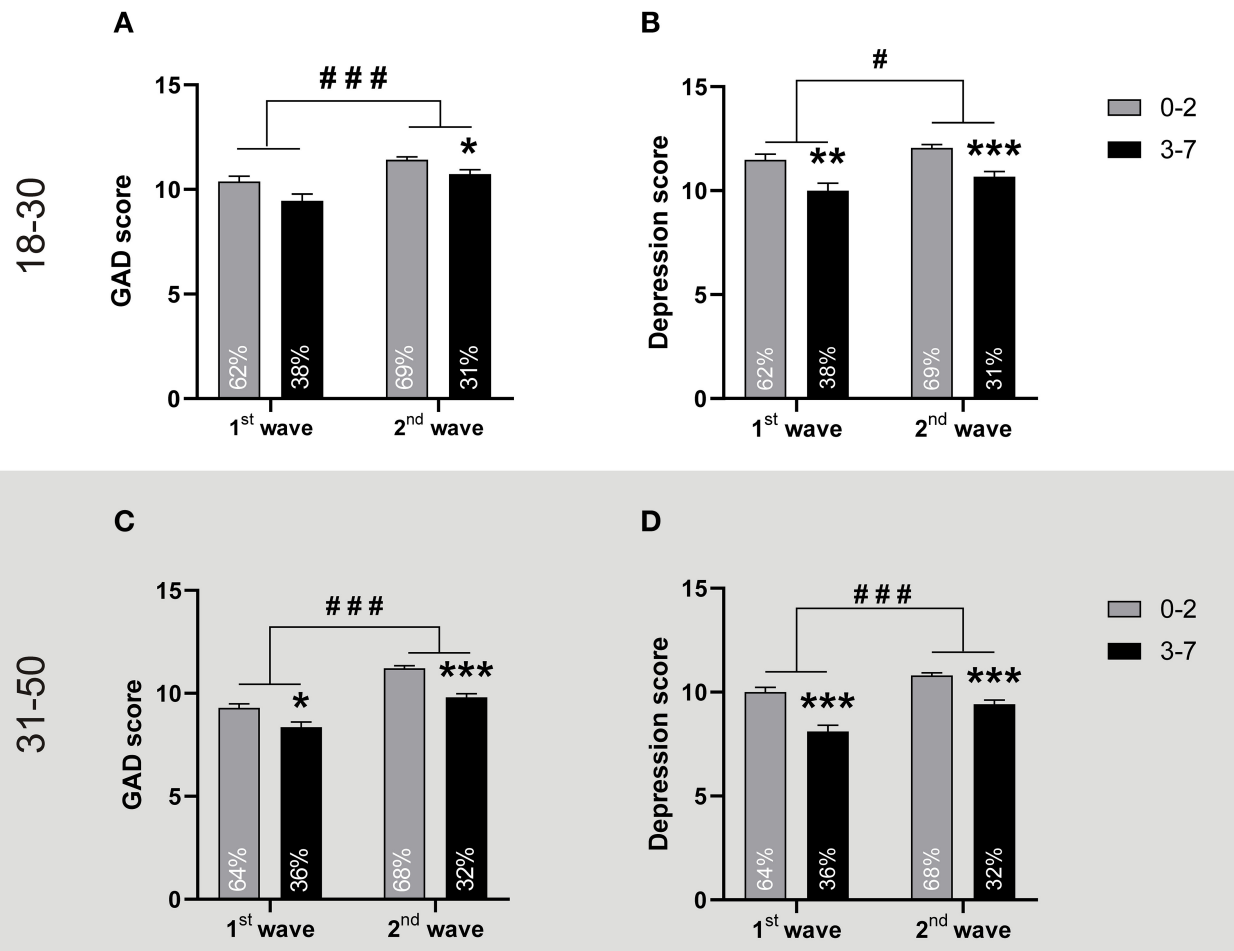
Practicing physical activity more than 2 days per week is associated with lower GAD and depression. **Top panel:** People aged between 18 and 30. The figures show the mean + SEM of **(A)** GAD scores and **(B)** Depression scores of young adults who practiced physical up to 2 days per week (0–2), or more (3–7), in the 1st wave and in the 2nd wave (*n* = 428; 257; 1,266 and 578, respectively). ^*^p < 0.05, ^**^p < 0.01, and ^***^p < 0.001 vs. 0–2 group and ^#^*p* < 0.05, ^###^*p* < 0.001 vs. 1st wave, after two-way ANOVA. Each bar also informs the percentage of the population studied in the wave that exercised with low or high frequency. For GAD scores: Cohen's f^2^ = 0.12 vs. 0–2 group in the 2^nd^ wave, $\eta_{\text{p}}^{2}$ = 0.004 for the physical activity frequency factor and $\eta_{\text{p}}^{2}$ = 0.009 for the wave factor. For depression scores Cohen's f^2^ = 0.15 and 0.23 vs. 0–2 group for the 1st and 2nd wave respectively; $\eta_{\text{p}}^{2}$ = 0.010 for the physical activity frequency factor and $\eta_{\text{p}}^{2}$ = 0.002 for the wave factor. The descriptive statistics are reported in Supplementary Table 5. **Bottom panel:** People aged between 31 and 50. The figures show the mean+SEM of **(C)** GAD scores and **(D)** Depression scores of adults who practiced physical activity up to 2 days per week (0–2), or more (3–7), in the 1st wave and in the 2nd wave (*n* = 542; 304; 1,870; and 862, respectively). ^*^p < 0.05, ^***^p < 0.001 vs. 0–2 group and ^###^*p* < 0.001 vs. 1st wave, after two-way ANOVA. Each bar also informs the percentage of the population studied in the wave that exercised with low or high frequency. For GAD scores: Cohen's f^2^ = 0.09 and 0.26 vs. 0–2 group in the 1st and 2nd wave respectively, $\eta_{\text{p}}^{2}$ = 0.008 for the frequency of physical activity factor and $\eta_{\text{p}}^{2}$ = 0.017 for the wave factor. For depression scores Cohen's f^2^ = 0.19 and 0.24 in the 1st and 2nd wave respectively; $\eta_{\text{p}}^{2}$ = 0.0134 for the frequency of physical activity factor and $\eta_{\text{p}}^{2}$ = 0.0056 for the wave factor. The descriptive statistics are reported in Supplementary Table 5.

Since performing physical activity has been associated with lower levels of GAD and depression (26) the survey also inquired the participants about the frequency they performed exercise. Then we clustered them into two groups, those who exercised up to 2 days per week (low frequency) and those who did it 3 days or more (high frequency). We observed that, despite the registered levels of GAD and depression increased both in young adults and in adults regardless of the frequency of physical activity, in all cases the high frequency of physical activity was associated with a lower anxiety (Figures 5A,B) and depression (Figures 5C,D). In fact, the percentage of participants exercising with low frequency almost doubled to those who did it with high frequency, regardless of the age range and the analyzed wave (Figure 5). Thus, while the changes in GAD and depression levels reported in the two waves were unrelated to the percentage of people performing more or less exercise, the group of persons performing frequent exercise were also the one with less anxiety and depression symptoms.

**Figure 6:**
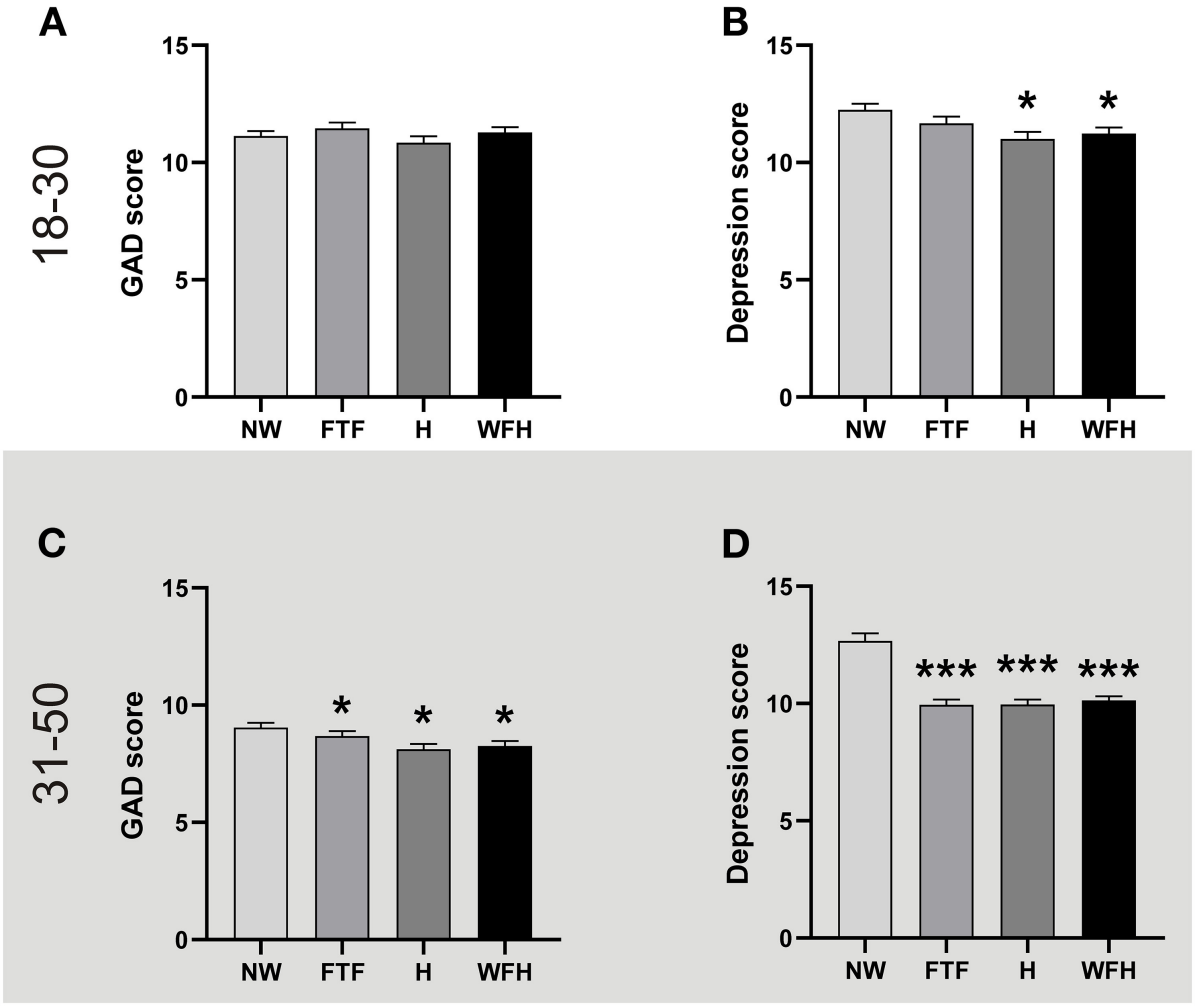
Working was associated with less depression during the second wave. The figures show the mean + SEM of GAD or Depression scores of non-working people (NW), and those who work face to face workers, hybrid modality (H), or from home (WFH). The descriptive statistics are reported in Supplementary Table 6. **Top panel:** People aged between 18 and 30. **(A)** GAD scores were equivalent regardless of the working status or modality. One-way ANOVA, *p* > 0.05. **(B)** Working was associated with lower depression levels except for FTF modality. ^*^p < 0.05, vs. NW, Kruskal-Wallis analysis after a one-way ANOVA (non-parametric); Cohen's f^2^ = 0.17 and 0.16, and vs. NW for H and WFH respectively; η^2^ = 0.0065. NW (*n* = 568), FTF (*n* = 451), H (*n* = 311), and WFH (*n* = 514). **Bottom panel:** People aged between 31 and 50. **(C)** Working was associated with lower GAD scores regardless of the modality. ^*^p < 0.05 vs. NW, Kruskal-Wallis analysis after ANOVA (non-parametric); Cohen's f^2^ = 0.06; 0.15 and 0.14 vs. NW for FTF, H and WFH respectively; η^2^ = 0.0064. **(D)** Working was associated with lower depression levels regardless of the modality. ^***^p < 0.001 vs. NW, Kruskal-Wallis analysis after ANOVA (non-parametric); Cohen's f^2^ = 0.32, 0.33 and 0.33 vs. NW for FTF, H and WFH respectively; η^2^ = 0.023. NW (*n* = 346), FTF (*n* = 630), H (*n* = 643), and WFH (*n* = 1,112).

Finally, considering that different working modalities were adopted since the beginning of the pandemic, we evaluated the effect of this variable on the anxiety and depression levels during the second wave, a moment when the change in the working modality was consolidated for most people in Argentina. In particular, we focused on the working status, that is, people who work or who do not work (non-working), and the work modality, that is, from home, face to face, or hybrid. As shown in Figure 6A the GAD of the young adult population was equivalent among non-workers and workers of the different modalities. On the contrary, adult working people were less anxious that non-working adult people, irrespective of their working modality (Figure 6C). This effect was more pronounced on the depression scores, where working adults, independently of the work modality, were less depressed than non-working ones (Figure 6D). A similar pattern, but less conspicuous and without evident effect of face-to-face work (*p* = 0.842), was observed in young adults (Figure 6B).

## Discussion

The most relevant results of this work indicate that subjects between 18 and 50 years old reported higher GAD and depression scores during the second wave of COVID-19 than those who did it after the first wave. In accordance with these results, the percentage of the population with a GAD and depression scores higher than moderate (equal to or higher than 10) increased during the second wave compared to the first one. Moreover, our results show a series of factors that help to mitigate the effect of GAD and depression in mental health. The most notorious factor was the effect of vaccination. Being vaccinated against COVID-19, was associated with lower levels of depression within the second wave. In contrast, being vaccinated did not alter GAD levels. Also, practicing physical activity more than twice a week was associated with reduced anxiety and depression in both the first and the second wave. Finally, lower GAD and depression levels were specifically identified during the second wave in adults of 31–50 years old who worked, either face-to-face or online, in contrast to those who did not work.

We start analyzing these results concerning others obtained in Argentina by different research groups. Reports from March 2020, at the start of the lockdown, informed in young people from 18 to 25 years old a prevalence of moderate to severe GAD of 35% (40). In April of the same year, reports from the general population evidenced a 31.8% incidence (4). Our data collected in November 2020 showed a prevalence of 46%, and in May 2021, at the second wave of infections, it was 56%. When analyzing the group aged between 25 and 44 years, the prevalence was 25% in March 2020 (40); in our group of adults (30–50 years old) in November of 2020, it was 42%, and in May of 2021, it was 57%. This information suggests that the percentage of adults between 18 and 50 years old who reported moderate to severe GAD symptoms increased steadily in the 15 months since the beginning of the lockdown in Argentina. The reports of moderate to severe depression of Argentinean young people show that its prevalence ranged between 40 and 60% from the beginning of the lockdown until May 2021 (40, 41). In March of the 2020th, its incidence in the population aged between 25 and 44 was around 30% (40). This value was similar to that obtained in the general population for April 2020 (4). Then, our results in the adult population for November 2020 showed 38% of incidence, and in May 2021, 44%. Together, the information of this age group evidences a slight but sustained increment of moderate to severe depression symptoms during the curse of the pandemic.

In the regional context, other countries that suffer from limited economies and resources for their health services also experienced the first COVID-19 outbreak at a comparable time course. A study compared GAD and depression symptoms carried out in seven of those Latin American countries (Argentina, Ecuador, Mexico, Paraguay, Uruguay, Colombia, and El Salvador) between June and September 2020, when they experienced their first wave of contagions. That research informed a prevalence of moderate to severe GAD and depression symptoms of 25–30%. The exception was Uruguay, with an incidence close to 10%. In the particular case of Argentina, at that time, these parameters were at 30% (42). On the other hand, Brazil, the most affected country by the pandemic of the region, reported between May and July 2020 an 81.90% prevalence of anxiety and 68% of depression, with moderate to severe symptoms (43). In Argentina, when the cases of contagion showed a sustained increase toward November 2020, we found a 40–50% prevalence for both parameters, and in May 2021, these values were between 45 and 57%. In Peru, another country in the region, the prevalence of depression was 44% in young adults, although in adults was 26% by May 2021 (44). Thus, according to these works, within the Latin American region, GAD and depression of the Argentine population increased through the pandemic, showing prevalence levels similar to some of its sister countries, except for Uruguay and Brazil, which respectively had lower and higher levels than our country.

As we mentioned before, the level of GAD was higher in the second wave of infections by COVID-19 in 2021 compared to the first one. It is known that GAD values correlate positively to depression values (45, 46). A study conducted in Australia in 2012 reports that 39% of individuals with GAD also meet criteria for depression and the authors found that comorbid depression and anxiety disorders occur in up to 25% of general practice patients (47). We obtained positive correlations between GAD and depression, regardless of the age range, in both outbreaks registered in the Metropolitan area of Buenos Aires. The correlation slopes during the second wave were equivalent to those of the first one. This indicates that the populations surveyed in both waves had equivalent relations and suggest that those factors associated with the higher GAD of the second wave are most likely those associated with the higher depression scores.

Our data reflects that being vaccinated is a key factor associated with a lower self-perception of depression. This may be due to a feeling of sanitary well-being, since people who accept to receive the vaccine for COVID-19 seek to protect their health, reduce the duration and severity of the disease and look for the outbreak to end (48). On the other hand, the most common reasons for vaccination refusal are related to fear of the vaccines' side effects, the lack of knowledge about their effectiveness, and distrust to vaccines developed abroad (48). Moreover, our data showed that people were more anxious in May 2021 (during Argentina second wave of contagious), when the vaccination campaign was accelerating, than 6 months before when vaccines were not yet available. This fact could be a predictor of higher tendency to receive COVID-19 vaccines, since greater anxiety, confidence in vaccines, and collective responsibility were associated with the request of vaccination (17). In particular, COVID-19-related anxiety, and fears of infection correlated positively with vaccine acceptance (15). Alternatively, the high degree of GAD reported in 2021 could also be due to vaccination hesitance. In this sense, it has been reported that having ambivalent attitudes toward vaccination are related to mental health morbidity such as depression, peritraumatic stress, but fundamentally triples the risk for anxiety (16). Thus, the rise in anxiety levels observed in May 2021 could be associated either to the acceptance or the hesitance to vaccination. It worth noticing the role of mass and social media toward vaccination hesitance by the distribution of sensationalistic and/or conspiracy theories (49, 50). Thus, beyond the negative effect of this infodemic on the public health by its effect on the vaccination campaigns, it may be also responsible of direct harm to people's mental health.

A study carried out at the end of February 2021 showed that in Latin American countries 8 out of 10 adults have vaccination intention and fear of its side effects. In Argentina, the people showed a 70–75% intention of vaccination with a lower frequency of fear of side effects than in other countries of the region (84.5%) (51). The constant recommendations from peers and healthcare providers, explaining the possible side effects against the benefits of being inoculated, and its frequency, may bring peace of mind to the population and increase the willingness to get vaccinated. In this way, more population shall accept the vaccines, bringing multiple benefits for the personal and public health. From one side, the direct personal and public benefit of protection toward physical by the use of vaccine, which demonstrated positive effects against the COVID-19 (52–56). And from the other side, contributing to the concomitant decrease in the levels of depression, as shown in this study. In fact, these psychological factors shape the antibody responses to vaccines. In this sense, fear of COVID-19 itself, stress, depression, loneliness, and social isolation can impair the vaccine's ability to confer immunity against the virus (18). In any case, as it has been observed that vaccinated people can contract and spread SARS-CoV-2, it is important to keep social distancing measures for preventing the progression of the viral infection during the mass vaccination campaign (57); even when social isolation can reduce direct and indirect effects of the vaccination on mental health of the people (21, 22).

Another parameter registered in the surveys was the frequency of physical activity performed by the population. We observed that those who exercised more than twice a week reported lower levels of GAD and depression than those who exercised less frequently. Our study revealed that GAD and depression levels of the studied population were higher in the second wave, regardless of the frequency that people exercised. However, within each wave, both surveys (November 2020 and May 2021) showed that the group that performed frequent physical activity also reported less anxiety and depression. Thus, while general changes between waves were independent of how much people exercised, our results show, in a consistent way and on a high number of participants, that frequent exercise benefits mental health, independently of its basal state in the population. So, recommendations to increase public awareness about the impact on mental health of interrupted daily routines should include regularizing existing positive routines, in particular, the practice of physical exercises that has been diminished in this pandemic (58, 59). In our study, the percentage of people who reported exercising more than twice a week decreased from 37 to 31% from the first wave of contagious to the second one. A research performed in Australia reported that half of responders declared a reduction in physical activity since the onset of the COVID-19 pandemic, which was likely a consequence of social distancing, the closure of usual exercise venues, or unwillingness to change previous exercise habits (25). As the result, during the lockdown the group of more sedentary people presented more anxiety and depression symptoms (60). This work did not delve into the neurobiological mechanisms by which physical activity affects the levels of anxiety and depression. However, it is worth noticing that they include the regulation of the hypothalamic-pituitary-adrenal axis, effects on the endogenous opioid system, and the increase of the brain-derived neurotrophic factor level, which also affect the reactivity to stress and mood (61, 62).

Finally, the consolidation of multiple working modalities during the pandemic let us wonder of their relation with the anxiety and the depression levels during the second wave. We observed that the group of adult working people had lower GAD and depression than people who did not work. Nevertheless, this was a work effect rather than a modality effect, as GAD and depression levels were equivalently low with independence of the working-modality (at home, face-to-face or hybrid). In the case of the young adults' group, a similar but less conspicuous association was observed only in the depression levels. Our data is in agreement with previous one, showing that active workers showed fewer depression symptoms than unemployed people did (63). The COVID-19 pandemic and lockdown seems particularly stressful for individuals without work who reported more mental health disorders (8).

In sum, our results show that the second wave of contagion in the Metropolitan area of Argentina surprised with higher GAD and depression levels than the first outbreak that occurred 6 months earlier. Being vaccinated was selectively associated with decreased levels of depression in adults between 18 and 50 years old. In addition, the regular practice of physical activity as well as working coupled to a reduced self-perception of anxiety and depression symptoms. Determining the factors that contribute to reduce the risk of GAD and depression is important at scientific, clinical and even political level. Particularly, in pandemic times when they over pass the normal population values this knowledge could be used to develop strategies, such as fostering physical activity practices, guarding the employment and accelerating the vaccination campaigns, in order to prevent further injuries to people's mental health.

## Strengths and Limitations

To our knowledge, this is the first study to analyze general anxiety and depressive symptoms in two consecutive COVID-19 outbreaks and relate them to the presence and administration of COVID19 vaccines in Argentina's largest urban conglomerate. Among other strengths, it is worth noticing the large sample size and its relative representability, for recruiting the participants of the general population. Another valuable aspect is the timing of the sampling during the two waves, in moments when none of them were vaccinated (first wave) and when only some of them were (second wave), which allowed to analyze non-vaccinated people between waves and vaccinated against non-vaccinated people within the same wave. Yet, this study has some limitations that require acknowledging. The online sampling, which allowed data recollection in lockdown periods, might be the major one. Most respondents were young to middle age people, highly educated, and actively involved in accessing to COVID19 and other scientific information. Therefore, self-selection bias could exist and affect the representativeness of the sample. Also, we decided to exclude social structure characteristics from our analysis, opening the possibility to a sociodemographic mismatch between the 1st and 2nd outbreak population of respondents. Yet, it is worth noticing that the same recruiting method (same social media from the same scientific communicators) was used in both outbreaks, thus reducing the possibility of this mismatch. Therefore, we tried to reach as many people as possible and be cautious in our conclusion. Regarding the sample size, more people responded to the second survey than the first one, probably indicating the rise of people's interest in this kind of studies during the development of the pandemics. Second, the number of women that responded to the survey was 3–4 times higher than the number proportion of men. Thus, the possibility exists that the mean in GAD and depression symptoms of the total population over represent the symptomology of women. As a positive aspect, it worth noticing than the behavior of GAD and depression symptomology, as well as their levels associated with vaccination, physical activity and working status were equivalent for women and men, with the notorious exception of young men population. Therefore, the behavior of the symptomology of the total population may well represent that of men and women. Third, we assessed the psychological impact on general anxiety disorder and depression through self-reported answers of the participants rather than clinical diagnosis by a physician. To minimize this limitation, we surveyed using the GAD-7 and PHQ-9 questionnaires. These are well-established tools for valid and efficient screening and assessing the severity of GAD and depression in clinical practice and research (2, 3, 5–10, 12, 17, 20, 21, 34–39). In addition, we think that increased or decreased symptomology in large samples may well represent the direct impact in general anxiety and depression disorders of the population. Fourth, the cross-sectional design adopted in this study implies that the association between GAD and depression symptomology with the different waves and vaccine inoculations is not necessarily causal. Also, other confounding factors associated with the vaccinated group of people may explain the decrease in depressive symptoms. Finally, this is a correlational study at group level. Therefore, the conclusions do not necessarily apply to a particular individual but reflect the possible risks and benefits for different groups in the population. Future longitudinal studies, at the individual level in other countries or regions, may help support our findings and rule out the possibility of ecological fallacy.

## Data Availability Statement

The raw data supporting the conclusions of this article will be made available by the authors, without undue reservation.

## Ethics Statement

The studies involving human participants were reviewed and approved by Ethics Council of the Life Sciences Department of the Instituto Tecnológico de Buenos Aires. The patients/participants provided their written informed consent to participate in this study.

## Author Contributions

DM, FB, HV, and JM contributed to the conception and the design of the study. FB, PB, and AB obtained the data. PB, AB, FM, VR, and CG organized the database, performed the statistical analyses, and sketched the figures. DM, HV, CK, FB, and JM oversight the statistical analyses and figures. DM and HV wrote the first draft of the manuscript. PB and AB wrote the first draft of the methods section. DM, HV, FB, CK, JM, PB, and AB contributed to the manuscript revision. All authors read and approved the final version of this manuscript.

## Conflict of Interest

The authors declare that the research was conducted in the absence of any commercial or financial relationships that could be construed as a potential conflict of interest.

## Publisher's Note

All claims expressed in this article are solely those of the authors and do not necessarily represent those of their affiliated organizations, or those of the publisher, the editors and the reviewers. Any product that may be evaluated in this article, or claim that may be made by its manufacturer, is not guaranteed or endorsed by the publisher.
