# Supplementary material for: Second Wave of COVID-19 Pandemic in Argentinian Population: Vaccination Is Associated With a Decrease in Depressive Symptoms
Source: Front Psychiatry. 2022 Jun 23;13:832352. doi: 10.3389/fpsyt.2022.832352 (PMC9259890; doi:10.3389/fpsyt.2022.832352)
Supplement: Supplementary file 1 [file Data_Sheet_1.pdf]

Supplementary statistics.

Supplementary Table 1 (Corresponds to figure 1).

Fig 1	Group	N	Mean	SEM	P-value	Analysis
a	1st<br>2nd	685<br>1844	10.04<br>11.20	0.1968<br>0.1168	<0,0001<br>***	Mann-Whitney U-test
b	1st<br>2nd	317<br>1027	46.28%<br>55.69%		<0,0001<br>***	Chi-squared
c	Men 1st<br>Men 2nd<br>Women 1st<br>Women 2nd	171<br>388<br>514<br>1456	8.901<br>9.155<br>10.41<br>11.75	0.393<br>0.247<br>0.225<br>0.129	Men 0.944<br>Women <0,0001 ***<br>Gender Factor <0,0001 # # #	Two-Way ANOVA
d	Men 1st<br>Men 2nd<br>Women 1st<br>Women 2nd	63<br>150<br>254<br>877	36.84%<br>38.66%<br>49.42%<br>60.23%		Men 0.286<br>Women <0,0001 ***	Chi-squared
e	1st<br>2nd	846<br>2732	8.954<br>10.78	0.1628<br>0.1004	<0,0001<br>***	Mann-Whitney U-test
f	1st<br>2nd	352<br>1564	41.61%<br>57.25%		<0,0001<br>***	Chi-squared
g	Men 1st<br>Men 2nd<br>Women 1st<br>Women 2nd	146<br>385<br>700<br>2347	7.315<br>8.87<br>9.296<br>11.09	0.361<br>0.26<br>0.179<br>0.11	Men 0.0099 **<br>Women <0,0001 ***<br>Gender Factor <0,0001 # # #	Two-Way ANOVA
h	Men 1st<br>Men 2nd<br>Women 1st<br>Women 2nd	36<br>158<br>312<br>1406	24.4%<br>41.04%<br>44.57%<br>59.91%		Men 0.0037 **<br>Women <0,0001 ***	Chi-squared

Table 1: Descriptive statistics corresponding to figure 1. Table shows for each panel (a-h, 1st column) and the experimental group (2nd column), the number of values (N), the mean or percentage of the population (in the case of Chi-squared tests), the standard error of the mean (SEM), the absolute P-value and the statistical analysis performed (Analysis).

Supplementary Table 2 (Corresponds to figure 2).

Fig 2	Group	N	Mean	SEM	P-value	Analysis
a	1st<br>2nd	685<br>1844	10.92<br>11.61	0.2225<br>0.1378	0.0061<br>**	Mann-Whitney U-test
b	1st<br>2nd	340<br>1003	49.64%<br>54.39%		0.0331<br>*	Chi-squared
c	Men 1st<br>Men 2nd<br>Women 1st<br>Women 2nd	171<br>388<br>514<br>1456	9.544<br>9.716<br>11.38<br>12.12	0.44<br>0.29<br>0.25<br>0.15	Men 0.988<br>Women 0.0635<br>Gender Factor <0,0001 # # #	Two-Way ANOVA
d	Men 1st<br>Men 2nd<br>Women 1st<br>Women 2nd	65<br>153<br>275<br>850	38.01%<br>39.43%<br>53.5%<br>58.38%		Men 0.759<br>Women 0.049 *	Chi-squared
e	1st<br>2nd	846<br>2732	9.316<br>10.37	0.1857<br>0.1113	<0,0001<br>***	Mann-Whitney U-test
f	1st<br>2nd	322<br>1214	38.06%<br>44.44%		0.0011<br>**	Chi-squared
g	Men 1st<br>Men 2nd<br>Women 1st<br>Women 2nd	146<br>385<br>700<br>2347	7.856<br>8.608<br>9.627<br>10.66	0.4045<br>0.295<br>0.206<br>0.1191	Men 0.522<br>Women <0,0001 ***<br>Gender Factor <0,0001 # # #	Two-Way ANOVA
h	Men 1st<br>Men 2nd<br>Women 1st<br>Women 2nd	47<br>129<br>314<br>1259	32.19%<br>33.51%<br>44.86%<br>53.64%		Men 0.7738<br>Women <0,0001 ***	Chi-squared

Table 2: Descriptive statistics corresponding to figure 2. Table shows for each panel (a-h, 1st column) and the experimental group (2nd column), the number of values (N), the mean or percentage of the population (in the case of Chi-squared tests), the standard error of the mean (SEM), the absolute P-value and the statistical analysis performed (Analysis).

Supplementary Table 3 (Corresponds to figure 3).

Fig 3	Group	N	Mean	SEM	P-value		Analysis
a	1°wave<br>Unvac<br>Vac	685<br>1538<br>303	10.04<br>11.14<br>11.50	0.1968<br>0.1268<br>0.2997	1°wave vs unvac<br><0,0001<br># # #	unvac vs vac<br>0.3368	Mann-Whitney U-test
b	1°wave<br>Unvac<br>Vac	514<br>1195<br>259	10.41<br>11.72<br>11.88	0.2251<br>0.1411<br>0.3157	1°wave vs unvac<br><0,0001<br># # #	unvac vs vac<br>0.8076	Mann-Whitney U-test
c	1°wave<br>Unvac<br>Vac	171<br>343<br>44	8.901<br>9.114<br>9.318	0.3932<br>0.2575<br>0.8332	1°wave vs unvac<br>0.5908	unvac vs vac<br>0.9348	Mann-Whitney U-test
d	1°wave<br>Unvac<br>Vac	846<br>2035<br>692	8.954<br>10.84<br>10.59	0.1628<br>0.1170<br>0.1964	1°wave vs unvac<br><0,0001<br># # #	unvac vs vac<br>0.2285	Mann-Whitney U-test
e	1°wave<br>Unvac<br>Vac	700<br>1709<br>633	9.296<br>11.19<br>10.82	0.1791<br>0.1261<br>0.2047	1°wave vs unvac<br><0,0001<br># # #	unvac vs vac<br>0.114	Mann-Whitney U-test
f	1°wave<br>Unvac<br>Vac	146<br>326<br>59	7.315<br>9.006<br>8.102	0.3618<br>0.2907<br>0.6115	1°wave vs unvac<br>0.0016<br># #	unvac vs vac<br>0.2407	Mann-Whitney U-test

Table 3: Descriptive statistics corresponding to figure 3. Table shows for each panel (a-h, 1st column) and the experimental group (2nd column), the number of participants (n), the mean, the standard error of the mean (SEM), the absolute P-value, and the statistical analysis performed (Analysis).

Supplementary Table 4 (Corresponds to figure 4).

Fig 4	Group	N	Mean	SEM	P-value		Analysis
a	1°wave<br>Unvac<br>Vac	685<br>1538<br>303	10.92<br>11.72<br>11.05	0.2225<br>0.1508<br>0.3393	1°wave vs unvac<br>0.0021<br># #	unvac vs vac<br>0.0680	Mann-Whitney U-test
b	1°wave<br>Unvac<br>Vac	514<br>1195<br>259	11.38<br>12.30<br>11.30	0.2549<br>0.1696<br>0.3560	1°wave vs unvac<br>0.0018<br># #	unvac vs vac<br>0.0115<br>*	Mann-Whitney U-test
c	1°wave<br>Unvac<br>Vac	171<br>343<br>44	9.544<br>9.691<br>9.614	0.4401<br>0.3042<br>1.017	1°wave vs unvac<br>0.6974	unvac vs vac<br>0.6021	Mann-Whitney U-test
d	1°wave<br>Unvac<br>Vac	846<br>2035<br>692	9.322<br>10.58<br>9.733	0.1855<br>0.1302<br>0.2131	1°wave vs unvac<br><0,0001<br># # #	unvac vs vac<br>0.0008<br>***	Mann-Whitney U-test
e	1°wave<br>Unvac<br>Vac	700<br>1709<br>633	9.627<br>10.90<br>9.997	0.2060<br>0.1407<br>0.228	1°wave vs unvac<br><0,0001<br># # #	unvac vs vac<br>0.0006<br>***	Mann-Whitney U-test
f	1°wave<br>Unvac<br>Vac	146<br>325<br>59	7.856<br>8.917<br>6.898	0.4045<br>0.3259<br>0.6242	1°wave vs unvac<br>0.0490<br>#	unvac vs vac<br>0.0119<br>*	Mann-Whitney U-test

Table 4: Descriptive statistics corresponding to figure 4. Table shows for each panel (a-h, 1st column) and the experimental group (2nd column), the number of participants (n), the mean, the standard error of the mean (SEM), the absolute P-value, and the statistical analysis performed (Analysis).

Supplementary Table 5 (Corresponds to figure 5).

Fig 5	Group	N	Mean	SEM	P-value		Analysis
a	(0-2) 1st<br>(3-7) 1st<br><br>(0-2) 2nd<br>(3-7) 2nd	428<br>257<br><br>1266<br>578	10.38<br>9.459<br><br>11.42<br>10.73	0.250<br>0.316<br><br>0.14<br>0.21	1st wave<br>0.0949<br><br>2nd wave<br>0.0327<br>*	Wave<br>Factor<br><0,0001<br># # #	Two-Way<br>ANOVA
b	(0-2) 1st<br>(3-7) 1st<br><br>(0-2) 2nd<br>(3-7) 2nd	428<br>257<br><br>1266<br>578	11.48<br>9.996<br><br>12.05<br>10.67	0.2786<br>0.3630<br><br>0.1648<br>0.2466	1st wave<br>0.0073<br>**<br><br>2nd wave<br><0,0001<br>***	Wave<br>Factor<br>0.0232<br>#	Two-Way<br>ANOVA
c	(0-2) 1st<br>(3-7) 1st<br><br>(0-2) 2nd<br>(3-7) 2nd	542<br>304<br><br>1870<br>862	9.290<br>8.355<br><br>11.22<br>9.807	0.209<br>0.255<br><br>0.120<br>0.178	1st wave<br>0.498<br>*<br><br>2nd wave<br><0,0001<br>***	Wave<br>Factor<br><0,0001<br># # #	Two-Way<br>ANOVA
d	(0-2) 1st<br>(3-7) 1st<br><br>(0-2) 2nd<br>(3-7) 2nd	542<br>304<br><br>1870<br>862	10.00<br>8.109<br><br>10.80<br>9.423	0.2337<br>0.2926<br><br>0.1330<br>0.1990	1st wave<br><0,0001<br>***<br><br>2nd wave<br><0,0001<br>***	Wave<br>Factor<br><0,0001<br># # #	Two-Way<br>ANOVA

Table 5: Descriptive statistics corresponding to figure 5. Table shows for each panel (a-h, 1st column) and the experimental group (2nd column), the number of participants (n), the mean, the standard error of the mean (SEM), the absolute P-value, and the statistical analysis performed (Analysis).

Supplementary Table 6 (Corresponds to figure 6).

Fig 6	Group	N	Mean	SEM	P-value	Analysis
a	NW<br>FTF<br>H<br>WFH	568<br>451<br>311<br>514	11.13<br>11.46<br>10.84<br>11.28	0.2127<br>0.2393<br>0.2747<br>0.2203	NW vs FTF<br>>0.9999<br>NW vs H<br>>0.9999<br>NW vs WFH<br>>0.9999	Kruskal-Wallis after One-Way ANOVA (non-parametric)
b	NW<br>FTF<br>H<br>WFH	568<br>451<br>311<br>514	12.25<br>11.67<br>11.01<br>11.23	0.2540<br>0.2799<br>0.3004<br>0.2664	NW vs FTF<br>0.8422<br>NW vs H<br>0.0499<br>*<br>NW vs WFH<br>0.0190<br>*	Kruskal-Wallis after One-Way ANOVA (non-parametric)
c	NW<br>FTF<br>H<br>WFH	346<br>630<br>643<br>1112	9.048<br>8.676<br>8.113<br>8.259	0.1967<br>0.2177<br>0.2307<br>0.2049	NW vs FTF<br>0.489<br>*<br>NW vs H<br>0.0194<br>*<br>NW vs WFH<br>0.0236<br>*	Kruskal-Wallis after One-Way ANOVA (non-parametric)
d	NW<br>FTF<br>H<br>WFH	346<br>630<br>643<br>1112	12.67<br>9.948<br>9.952<br>10.13	0.3176<br>0.2262<br>0.2190<br>0.1755	NW vs FTF<br><0,0001<br>***<br>NW vs H<br><0,0001<br>***<br>NW vs NFH<br><0,0001<br>***	Kruskal-Wallis after One-Way ANOVA (non-parametric)

Table 6: Descriptive statistics corresponding to figure 6. Table shows for each panel (a-h, 1st column) and the experimental group (2nd column), the number of participants (n), the mean, the standard error of the mean (SEM), the absolute P-value, and the statistical analysis performed (Analysis).

Supplementary Table 7 (Corresponds to figure 1).

Fig 1	Group	Kolmogorov-Smirnov	Homoscedasticity test
a	1st<br>2nd	p<0,0001<br>***<br>p<0,0001<br>***	p=0.3903<br>F-test
c	Men 1st<br>Men 2nd<br>Women 1st<br>Women 2nd	p=0.0049<br>**<br>p<0,0001<br>***<br>p<0,0001<br>***<br>p<0,0001<br>***	p=0.6028<br>Bartlett test
e	1st<br>2nd	p<0,0001<br>***<br>p<0,0001<br>***	p=0.0003<br>***<br>F-test
g	Men 1st<br>Men 2nd<br>Women 1st<br>Women 2nd	p<0.0001<br>***<br>p<0,0001<br>***<br>p<0,0001<br>***<br>p<0,0001<br>***	p=0.0019<br>**<br>Bartlett test

Table 7: Normality and homocedasticity tests for figure 1. Table shows the corresponding panels (a-g, 1st column) and the experimental group (2nd column), the normality and homocedasticity test.

Supplementary Table 8 (Corresponds to figure 2).

Fig 2	Group	Kolmogorov-Smirnov	Homoscedasticity test
a	1st<br>2nd	p<0,0001<br>***<br>p<0,0001<br>***	p=0.6271<br>F-test
c	Men 1st<br>Men 2nd<br>Women 1st<br>Women 2nd	p=0.009<br>***<br>p<0,0001<br>***<br>p<0,0001<br>***<br>p<0,0001<br>***	p=0.1712<br>Bartlett test
e	1st<br>2nd	p<0,0001<br>***<br>p<0,0001<br>***	p=0.0084<br>**<br>F-test
g	Men 1st<br>Men 2nd<br>Women 1st<br>Women 2nd	p<0.0001<br>***<br>p<0,0001<br>***<br>p<0,0001<br>***<br>p<0,0001<br>***	p=0.0205<br>*<br>Bartlett test

Table 8: Normality and homocedasticity tests for figure 2. Table shows the corresponding panels (a-g, 1st column) and the experimental group (2nd column), the normality and homocedasticity test.

Supplementary Table 9 (Corresponds to figure 3).

Fig 3	Group	Kolmogorov-Smirnov	Homoscedasticity test
a	1°wave<br>Unvac<br>Vac	p<0,0001 ***<br>p<0,0001 ***<br>p<0,0001 ***	p=0.3863 Bartlett test
b	1°wave<br>Unvac<br>Vac	p<0,0001 ***<br>p<0,0001 ***<br>p=0,0003 ***	p=0.4099 Bartlett test
c	1°wave<br>Unvac<br>Vac	p=0,0049 **<br>p<0,0001 ***<br>P=0,0430 *	p=0.2810 Bartlett test
d	1°wave<br>Unvac<br>Vac	p<0,0001 ***<br>p<0,0001 ***<br>p<0,0001 ***	p=0.0010 ** Bartlett test
e	1°wave<br>Unvac<br>Vac	p<0,0001 ***<br>p<0,0001 ***<br>p<0,0001 ***	p=0.0112 * Bartlett test
f	1°wave<br>Unvac<br>Vac	p<0,0001 ***<br>p<0,0001 ***<br>p<0,0001 ***	p=0.0347 * Bartlett test

Table 9: Normality and homocedasticity tests for figure 3. Table shows the corresponding panels (a-g, 1st column) and the experimental group (2nd column), the normality and homocedasticity test.

Supplementary Table 10 (Corresponds to figure 4).

Fig 4	Group	Kolmogorov-Smirnov	Homoscedasticity test
a	1°wave<br>Unvac<br>Vac	p<0,0001<br>***<br>p<0,0001<br>***<br>P=0.0010<br>***	p=0.8995<br>Bartlett test
b	1°wave<br>Unvac<br>Vac	p<0,0001<br>***<br>p<0,0001<br>***<br>0.0099<br>***	p=0.8570<br>Bartlett test
c	1°wave<br>Unvac<br>Vac	p=0.0009<br>***<br>p<0,0001<br>***<br>P=0.0145<br>*	p=0.2568<br>Bartlett test
d	1°wave<br>Unvac<br>Vac	p<0,0001<br>***<br>p<0,0001<br>***<br>p<0,0001<br>***	p=0.0114<br>*<br>Bartlett test
e	1°wave<br>Unvac<br>Vac	p<0,0001<br>***<br>p<0,0001<br>***<br>p<0,0001<br>***	p=0.1085<br>Bartlett test
f	1°wave<br>Unvac<br>Vac	P=0.0001<br>***<br>p<0,0001<br>***<br>p<0,0001<br>***	p=0.0106<br>*<br>Bartlett test

Table 10: Normality and homocedasticity tests for figure 4. Table shows the corresponding panels (a-g, 1st column) and the experimental group (2nd column), the normality and homocedasticity test.

Supplementary Table 11 (Corresponds to figure 5).

Fig 5	Group	Kolmogorov-Smirnov	Homoscedasticity test
a	(0-2) 1st<br>(3-7) 1st<br><br>(0-2) 2nd<br>(3-7) 2nd	p<0,0001<br>***<br>p<0,0001<br>***<br>p<0,0001<br>***<br>p<0,0001<br>***	p=0.7851<br>Bartlett test
b	(0-2) 1st<br>(3-7) 1st<br><br>(0-2) 2nd<br>(3-7) 2nd	p<0,0001<br>***<br>p<0,0001<br>***<br>p<0,0001<br>***<br>p<0,0001<br>***	p=0.9374<br>Bartlett test
c	(0-2) 1st<br>(3-7) 1st<br><br>(0-2) 2nd<br>(3-7) 2nd	p<0,0001<br>***<br>p<0,0001<br>***<br>p<0,0001<br>***<br>p<0,0001<br>***	p=0.0016<br>**<br>Bartlett test
d	(0-2) 1st<br>(3-7) 1st<br><br>(0-2) 2nd<br>(3-7) 2nd	p<0,0001<br>***<br>p<0,0001<br>***<br>p<0,0001<br>***<br>p<0,0001<br>***	p=0.0154<br>*<br>Bartlett test

Table 11: Normality and homocedasticity tests for figure 5. Table shows the corresponding panels (a-g, 1st column) and the experimental group (2nd column), the normality and homocedasticity test.

Supplementary Table 12 (Corresponds to figure 6).

Fig 6	Group	Kolmogorov-Smirnov	Homoscedasticity test
a	NW	p=0,0042<br>**	p=0.7927<br>Bartlett test
	FTF	p<0,0001<br>***	
	H	0,0092<br>**	
	WFH	p<0,0001<br>***	
b	NW	p<0,0001<br>***	p=0.0440<br>*<br>Bartlett test
	FTF	p<0,0001<br>***	
	H	0,0002<br>***	
	WFH	p<0,0001<br>***	
c	NW	p<0,0001<br>***	p=0.1945<br>Bartlett test
	FTF	p<0,0001<br>***	
	H	0,0001<br>***	
	WFH	p<0,0001<br>***	
d	NW	P=0,0160<br>*	p=0.4022<br>Bartlett test
	FTF	p<0,0001<br>***	
	H	P=0,0002<br>***	
	WFH	p<0,0001<br>***	

Table 11: Normality and homocedasticity tests for figure 4. Table shows the corresponding panels (a-g, 1st column) and the experimental group (2nd column), the normality and homocedasticity test.

Supplementary Materials and Methods

Next, we provide the survey conducted during the second wave. The survey implemented during the first wave was identical except for the lack of point 8 because vaccines were unavailable at that moment. It is worth noticing that the survey is provided in Spanish because it was conducted in this language. It starts on the next page under the title “Encuesta”.

Encuesta

Somos un grupo interdisciplinario de investigadores e investigadoras de la Universidades de Buenos Aires, del ITBA y de la UNICEN, los cuales estamos intentando comprender cómo nos afecta emocionalmente esta pandemia. La encuesta demora unos pocos minutos y es muy importante que la completes con el mayor grado de sinceridad posible.

Tu respuesta nos ayuda muchísimo a generar estrategias para mitigar las consecuencias de la pandemia.

*Obligatorio

1. La participación en esta encuesta es VOLUNTARIA. La información solicitada en el present[illegible] cuestionario es totalmente CONFIDENCIAL, solo será utilizada para fines cientificos. Una v[illegible] concluida, los datos de ANONIMIZAN de manera automática.

Marca solo un óvalo.

( ) Doy mi consentimiento para que los datos obtenidos a partir de la encuesta realizada en noviembre y mayo sean utilizados con fines científicos y de investigación.

Datos Personales

2. Nombre *

Los datos son CONFIDENCIALES. El nombre y apellido solamente se utilizan para MATCHEAR con la prueba de CREATIVIDAD.

____

3. Apellido *

Los datos son CONFIDENCIALES. El nombre y apellido solamente se utilizan para MATCHEAR con la prueba de CREATIVIDAD.

____

4. Provincia

Marca solo un óvalo.

- CABA
- GBA
- Buenos Aires (excepto AMBA)
- Catamarca
- Chaco
- Chubut
- Cordoba
- Corrientes
- Entre Rios
- Formosa
- Jujuy
- La Pampa
- La Rioja
- Mendoza
- Neuquen
- Rio Negro
- Salta
- San Juan
- San Luis
- Santa Cruz
- Santa Fe
- Santiago del Estero
- Tierra del fuego e Islas de Atlántico Sur
- Tucuman
- Misiones

5. Edad *

Marca solo un óvalo.

- 18

- [ ] 19
- [ ] 20
- [ ] 21
- [ ] 22
- [ ] 23
- [ ] 24
- [ ] 25
- [ ] 26
- [ ] 27
- [ ] 28
- [ ] 29
- [ ] 30
- [ ] 31
- [ ] 32
- [ ] 33
- [ ] 34
- [ ] 35
- [ ] 36
- [ ] 37
- [ ] 38
- [ ] 39
- [ ] 40
- [ ] 41
- [ ] 42
- [ ] 43
- [ ] 44
- [ ] 45
- [ ] 46
- [ ] 47
- [ ] 48
- [ ] 49
- [ ] 50
- [ ] 51
- [ ] 52

- 53
- 54
- 55
- 56
- 57
- 58
- 59
- 60
- 61
- 62
- 63
- 64
- 65
- 66
- 67
- 68
- 69
- 70
- 71
- 72
- 73
- 74
- 75
- 76
- 77
- 78
- 79
- 80

6. Género *

Marca solo un óvalo.

- Mujer
- Varón
- Prefiero no decirlo

Vacunación COVID-19

7. Estas vacunada/o contra el COVID-19? *

Marca solo un óvalo.

- Si
- No
- Prefiero no decirlo

Vacunación COVID-19

8. En que mes te vacunaste ?

Marca solo un óvalo.

- Diciembre 2020
- Enero 2021
- Febrero 2021
- Marzo 2021
- Abril 2021
- Mayo 2021
- Prefiero no decirlo

Trabajo

9. ¿Estás trabajando actualmente? *

Marca solo un óvalo.

- Sí *Ir a la pregunta 10*
- No *Ir a la pregunta 11*

Trabajo

10. ¿Bajo que modalidad estás trabajando? *

Marca solo un óvalo.

- Virtual
- Presencial
- Híbrido entre ambos

Datos personales

11. ¿Cuántas veces a la semana realizas actividad física? *

Marca solo un óvalo.

- 0
- 1
- 2
- 3
- 4
- 5
- 6
- 7

En las ULTIMAS 2 SEMANAS, ¿con qué frecuencia has estado molesta/o por los siguientes problemas?

12. Sentirte nerviosa/o, ansiosa/o o al borde *

Marca solo un óvalo.

- Para nada
- Pocos dias
- Mas de la mitad de los dias
- Casi todos los dias

13. No poder detenerte o controlar las preocupaciones *

Marca solo un óvalo.

- Para nada
- Pocos dias
- Mas de la mitad de los dias
- Casi todos los dias

14. Preocuparte demasiado por distintas cosas *

Marca solo un óvalo.

- Para nada
- Pocos dias
- Mas de la mitad de los dias
- Casi todos los dias

15. Problemas para relajarte *

Marca solo un óvalo.

- Para nada
- Pocos dias
- Mas de la mitad de los dias
- Casi todos los dias

16. Estar tan inquieta/o que es difícil quedarse quieta/o *

Marca solo un óvalo.

- Para nada
- Pocos dias
- Mas de la mitad de los dias
- Casi todos los dias

17. Volverse fácilmente molesta/o o irritable *

Marca solo un óvalo.

- Para nada
- Pocos dias
- Mas de la mitad de los dias
- Casi todos los dias

18. Sentir miedo de que algo horrible pueda pasar *

Marca solo un óvalo.

- Para nada
- Pocos dias
- Mas de la mitad de los dias
- Casi todos los dias

19. Si marcaste algún problema, ¿cuánta dificultad te generaron para estudiar, hacer tareas d la casa o relacionarte con otras personas?

Marca solo un óvalo.

- Ninguna dificultad
- Algo difícil
- Muy difícil
- Extremadamente difícil

En las ULTIMAS 2 SEMANAS, ¿con qué frecuencia has estado molesta/o por los siguientes problemas?

20. Poco interés o placer en hacer cosas *

Marca solo un óvalo.

- Para nada
- Pocos dias
- Más de la mitad de los días
- Casi todos los días

21. Sentirte decaído/a,sin esperanzas *

Marca solo un óvalo.

- Para nada
- Pocos dias
- Más de la mitad de los días
- Casi todos los días

22. Dificultad para dormirte o mantener el sueño, o dormir demasiado *

Marca solo un óvalo.

- Para nada
- Pocos dias
- Más de la mitad de los días
- Casi todos los días

23. Sentirte cansado/a o con poca energía *

Marca solo un óvalo.

- Para nada
- Pocos dias
- Más de la mitad de los días
- Casi todos los días

24. Tener poco apetito o comer de más *

Marca solo un óvalo.

- Para nada
- Pocos dias
- Más de la mitad de los días
- Casi todos los días

25. Sentirte mal acerca de vos mismo/a, que sos un fracaso o que te decepcionaste a vos mismo/a o a tu familia

Marca solo un óvalo.

- Para nada
- Pocos dias
- Más de la mitad de los días
- Casi todos los días

26. Dificultad para concentrarte en las cosas, como leer el diario o ver la televisión *

Marca solo un óvalo.

- Para nada
- Pocos dias
- Más de la mitad de los días
- Casi todos los días

27. Moverte o hablar tan lentamente que otras personas lo puedan haber notado. O al contrario, estar tan inquieto/a o nervioso/a que te estuviste moviendo mucho más de lo usual

Marca solo un óvalo.

- Para nada
- Pocos dias
- Más de la mitad de los días
- Casi todos los días

28. Pensamientos sobre que seria mejor que no este o deseo de lastimarme *

Marca solo un óvalo.

- Para nada
- Pocos dias
- Más de la mitad de los días
- Casi todos los días
- Prefiero no decirlo

29. Si marcaste alguno de los problemas anteriores, ¿cuán más difícil han hecho estos problemas tu trabajo, ocuparte de las cosas en casa o interactuar con otras personas?

Marca solo un óvalo.

- Ninguna dificultad
- Algo más difícil
- Muy difícil
- Extremadamente difícil

Google no creó ni aprobó este contenido.

Google Formularios
